# Unexpected lessons from the neglected: How defective viral genomes became important again

**DOI:** 10.1371/journal.ppat.1007450

**Published:** 2019-01-10

**Authors:** Carolina B. López

**Affiliations:** Department of Pathobiology, School of Veterinary Medicine, University of Pennsylvania, Philadelphia, Pennsylvania, United States of America; The Fox Chase Cancer Center, UNITED STATES

The deeper we dig, the more we realize that there is much more to a virus infection than is initially apparent. Historically, major research efforts have been invested in understanding the biology and impact of the standard fully replicative virus during infection. This is the virus that spreads, infects additional individuals, and evolves to become increasingly more successful in the process. Because of its ability to propagate the infection, this is also the version of the virus that we target with antivirals and therapeutics. However, we now appreciate that virus variants and additional secondary products of virus replication play a critical role in determining the quality of the host immune response and the outcome of the infection, including the survival of both the virus and its host. Importantly, increased appreciation of the role of incomplete viral replication products in shaping the infection outcome is revealing new mechanisms that could be targeted to diminish virus pathogenesis.

My laboratory studies the effect of defective viral genomes in modulating virus pathogenesis. In addition, we are interested in understanding the role of these types of genomes in shaping the long-term virus-host interaction. Defective viral genomes were first observed in the 1940s as incomplete forms of influenza virus, and since then, they have been found in every scrutinized virus. However, after decades in the spotlight, the scientific community lost interest in defective viral genomes, in part because it became a nonfundable subject due to lack of evidence for their role in natural infections. Regrettably, basic research to study how defective viral genomes contribute to shaping infection dynamics and outcomes was delayed, and the potential to harness defective viral genomes to improve human and animal health was not appreciated until recently.

So, how did I end studying what others had dismissed as unimportant and artifactual byproducts of virus replication?

Before moving to the United States, I trained in Chile as a biochemist and immunologist. As an undergraduate student, I worked in an immunology laboratory studying contact dermatitis to urushiols, the compound in poison ivy that causes a severe allergic reaction in many people. During that time, I became more and more intrigued by viruses, as I realized how spectacularly efficient and resourceful they are. In graduate school, I settled in a lab that studied antiviral immunity. I remember nervously approaching Tom Moran to ask about joining his group before I had even set foot in the lab for my rotation. To my surprise, he immediately said yes. After working for months on testing a hypothesis that proved incorrect, I began to study how dendritic cells from the immune system responded to viral infections. This was a hot topic at the time, because dendritic cells were being recognized as pivotal players at the onset of the immune response, and Toll-like receptors were identified as the primary cellular sensors of microbes. Our data comparing the immune response to infection with two strains of a mouse virus (Sendai virus [SeV]) revealed that Toll-like receptors were not the main initiators of the immune response against this virus (we now know that SeV is sensed by retinoic acid-inducible gene-I [RIG I]-like receptors) and that an unknown viral component conferred strong ability to activate dendritic cells and initiate the immune response to one strain of the virus but not the other. I wanted to figure out what was driving differences in the ability of these two viral strains to initiate the antiviral immune response, and there was no better place within my geographical limitations to continue with this work, so I decided to continue in Tom’s lab for my postdoc (a very unpopular decision, I have to admit!) and then as an assistant professor on the research track. Together with Jacob Yount, then a graduate student, we tested whether mutations in the virus or differences in the activity of viral-encoded molecules that antagonize the cellular immune response explained our observations. The exciting result only came when we evaluated the role of defective particles that contain defective viral genomes. Adding defective particles to the poorly immunostimulatory virus turned it into one with strong immunostimulatory activity. Similarly, removing these particles from the highly immunostimulatory strain crippled its ability to activate dendritic cells. This result was one of the most exciting and memorable of my career so far. I remember thinking: how cool! No one cares about these types of viral genomes anymore and yet these data show that we can learn so much about how viruses trigger effective antiviral immunity by studying them!

I founded my laboratory in September 2010 based on that initial experiment. Since then, we have demonstrated that defective viral genomes have an essential role in determining the infection outcome during experimental infection with a number of respiratory viruses. Additionally, we have shown that defective viral genomes are present in humans when infected with respiratory syncytial virus, a common natural human respiratory viral infection. Through our work, we have enhanced our understanding of the short- and long-term impact that defective viral genomes have in infected cells and the molecular mechanisms involved. Most importantly, the field is now reinvigorated with a number of laboratories around the globe studying defective viral genomes of many different viruses. It is my hope that these studies, which were founded on curiosity, lead to a much more accurate understanding of the interaction between viruses and their host and eventually open new avenues for controlling pathogenic viral infections.

**Image 1:**
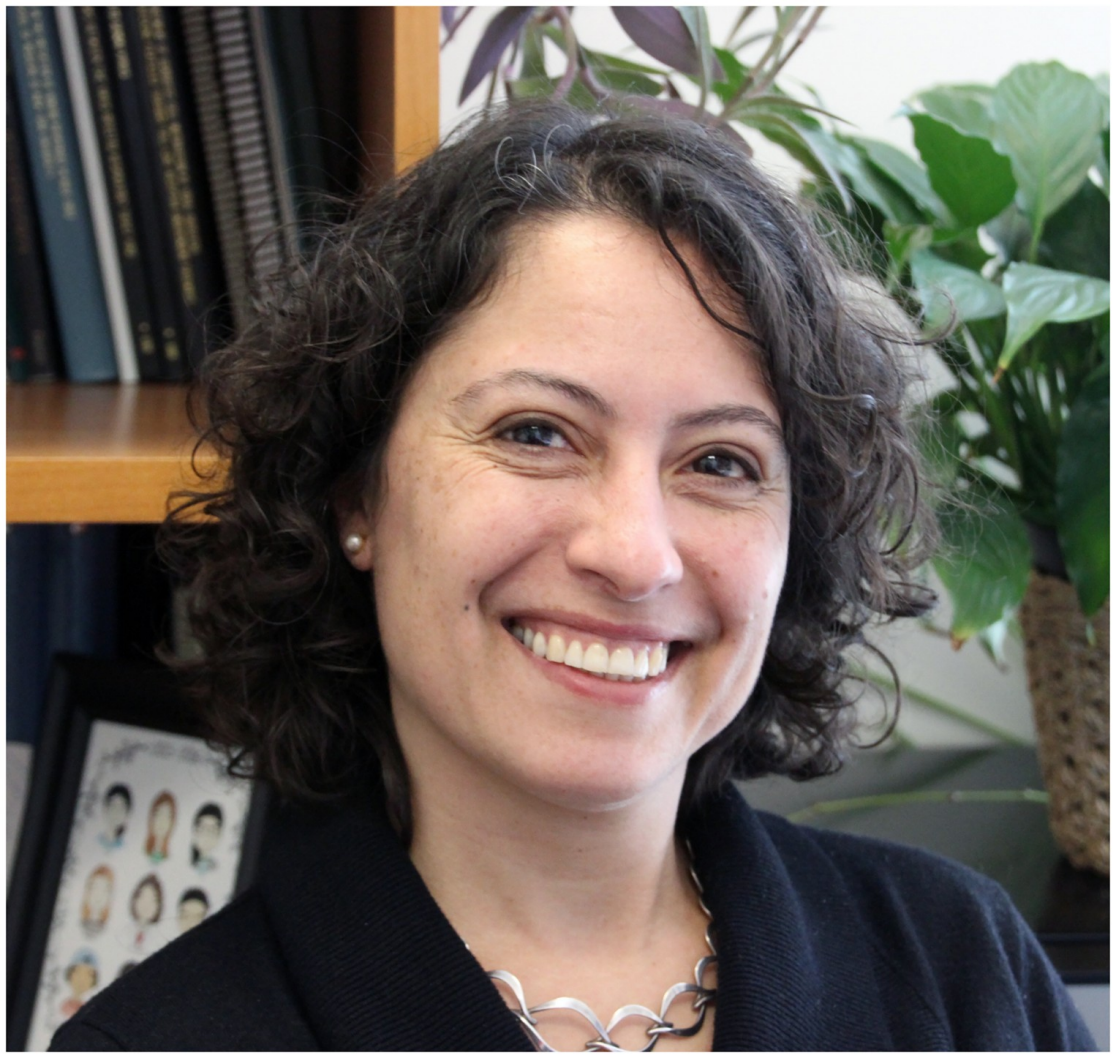
Carolina B. López.

